# Frail Phenotype and Disability Prediction in Community-Dwelling Older People: A Systematic Review and Meta-Analysis of Prospective Cohort Studies

**DOI:** 10.1097/jnr.0000000000000299

**Published:** 2019-05-20

**Authors:** Shu-Fang CHANG, Chih-Ling CHENG, Hsiang-Chun LIN

**Affiliations:** 1 PhD, RN, Professor, School of Nursing, College of Nursing, National Taipei University of Nursing and Health Sciences; 2 MSN, RN, Nurse, U Sweet Postpartum Nursing Home; 3 MSN, RN, Nurse, Medical Intensive Care Unit, MacKay Memorial Hospital.

**Keywords:** frailty, disability, systematic review, meta-analysis

## Abstract

**Background:**

The World Health Organization has identified frailty as a crucial factor affecting successful aging. Systematic literature reviews have yet to focus on the relationship between stages of frailty and disability in community-dwelling older adults.

**Purpose:**

The purpose of this study was to investigate the relationships between various frailty types and disability in community-dwelling older adults and to explore how various frailty criteria have been used to assess disability in this population.

**Methods:**

A systematic literature review and meta-analysis were conducted on articles from the following databases: Cochrane Library, CINAHL, PubMed, and Ovid. Database search criteria included articles that were published between January 2001 and July 2017 and study samples that included community-dwelling adults aged 60 years and older. We excluded studies that were conducted in institutions or hospitals and experimental studies on frailty. Two reviewers independently assessed eligibility and extracted data. A random-effects model was used to analyze the literature and to calculate the pooled disability of frailty.

**Results:**

In total, nine studies with a total sample of 32,998 participants that recorded 8,666 disabilities and a mean follow-up time of 30.4 months (*SD* = 29.26) were pooled for the meta-analysis. Using various indicators to predict the risk of disability compared with robust older adults, those with frailty faced a higher risk of disability, followed by older adults at risk of frailty.

**Conclusions/Implications for Practice:**

Frailty is a crucial health consideration among older adults. Those who are frail have the highest relative risk of disability, followed by those at risk of frailty. Early assessment of frailty may effectively prevent the occurrence of frailty-induced disability in older adults.

## Introduction

In 2002, the United Nations appealed to all countries to prioritize aging-related policy-making and action strategies (United Nations, 2009). Subsequently, the World Health Organization (WHO) advocated the importance of active aging, which has become a core concept in geriatric health policy in various countries (Department of Noncommunicable Disease Prevention and Health Promotion, WHO, 2002). According to the United Nations, 15% of people worldwide live with disabilities. More than 46% of older adults (aged 60 years or older) experience disability, and more than 250 million experience moderate to severe disability (United Nations, 2015). Aging is a global trend, and older adults are at a high risk for disability. The United Nations has urged various countries to review and explore disability in older adults. Virués-Ortega et al. (2011) investigated the current disability status of people aged 75 years and older and found that 39.17% ± 2.18% experienced mild disability, 15.31% ± 1.61% experienced moderate disability, and 10.14% ± 1.35% experienced severe disability. Overall, 65.62% of these older adults experienced mild or higher levels of disability. Keshari and Shankar (2017) investigated community-dwelling older adults and found disability at a prevalence of 53.6% (95% confidence interval [CI] [49.67, 57.5]). Gupta, Mani, Rai, Nongkynrih, and Gupta (2014) also investigated community-dwelling older adults and found disability at a prevalence of 37.4% (95% CI [34.2, 40.7]). According to the aforementioned studies, a high proportion of community-dwelling older adults experience disability. The older population is a highly heterogeneous group. Although many older adults experience illness and disability, people of the same age do not necessarily have the same health conditions. Because of this heterogeneity, geriatric health assessment is a great challenge. Frailty has been considered as a precursor to functional deterioration in older adults and as a stage between independent living and death (Chang & Lin, 2015).

The WHO (2002) indicated that frailty is a crucial factor related to successful aging. Frailty can affect the functions and quality of daily life in older adults. According to previous studies, the prevalence of frailty among people aged 65 years or older is between 5.8% and 35% (Kulmala, Nykänen, Mänty, & Hartikainen, 2014; Romero-Ortuno, Walsh, Lawlor, & Kenny, 2010); approximately 18.8%–50.9% can be considered at risk of frailty (Chang et al., 2014; Romero-Ortuno et al., 2010; Santos-Eggimann, Cuénoud, Spagnoli, & Junod, 2009). Numerous studies have indicated that frailty reduces activity level and quality of life (Chang & Wen, 2016), impairs cognitive function (Sánchez-García et al., 2014), and increases the likelihood of disability. Furthermore, disability in older adults reduces quality of life and increases the workload of and financial burden on caregivers (Chang & Chiu, 2015).

As indicated in previous studies, compared with robust older adults, frail older adults face a higher risk of disability (Abizanda et al., 2013; Macklai, Spagnoli, Junod, & Santos-Eggimann, 2013; Sánchez-García et al., 2014). Notably, controversy exists regarding the relative risks of disability between frail older adults and those at risk of frailty and between not yet frail and robust older adults.

Some studies have indicated that frail older adults have the highest risk of disability compared with older adults who are either at risk for frailty or still robust (Al Snih et al., 2009; Ensrud et al., 2008, 2009; Kiely, Cupples, & Lipsitz, 2009; Sánchez-García et al., 2014). However, other studies have come to different conclusions, finding no significantly higher risk of disability among these groups (Abizanda et al., 2013; Ávila-Funes et al., 2009; Bandeen-Roche et al., 2006; Ensrud et al., 2008, 2009). These contradictory findings make it difficult for nursing professionals to clearly determine whether frailty is related to disability or which frailty phenotypes exhibit higher risks of disability and to propose appropriate strategies to prevent disability for this high-risk group. Therefore, a meta-analysis should be conducted to examine the disparate assertions in the literature.

In addition, studies have used different indicators of frailty to assess disability in community-dwelling older adults. However, no studies have compared various indicators of frailty in predicting disability incidence among this population. The lack of consensus regarding frailty-related assessments is a problem for health providers. On the basis of these reasons, further analysis of the relationship between disability and the various stages of frailty, and the effect of frailty criteria on the relationship between stages of frailty and disability, is necessary.

The results of this study may help nursing staff to understand whether frailty predicts disability, use valid assessment indicators to identify community-dwelling older adults with frailty at an early stage, and develop effective prevention strategies to reduce the occurrence of disability in older adults with frailty.

### Aims

A systematic review and meta-analysis were conducted to explore whether frailty predicts the occurrence of disability in community-dwelling older adults. In addition, the comparative effectiveness of various indicators of frailty in predicting disability in community-dwelling older adults was examined.

## Methods

### Data Sources and Search Strategy

The Cochrane Library, CINAHL, PubMed, and Ovid databases were searched for articles containing the keywords “frailty” or “frail” and “disability” and “BADL” or “IADL” and “older people” or “older” or “geriatric” or “senior.” In related studies published before 2000, frailty mostly referred to comorbidity or disability. The three terms were often used interchangeably until 2001, when Fried et al. (2001) clearly differentiated frailty from comorbidity and disability. Therefore, data were collected for this study from articles published between January 2001 and July 2017.

### Inclusion and Exclusion Criteria

Articles that addressed the three stages of frailty (frail, at risk of frailty, and robust), used a prospective study design (because the aim of this study was to explore whether frailty predicts disability), included a follow-up period of more than 6 months, and targeted community-dwelling older adults aged 60 years or more were included. The adjusted or unadjusted hazard ratio (*HR*) was applied to the analysis results. Letters to the editor, book chapters, theses, and dissertations were excluded. In addition, studies that were conducted in institutions or hospitals and experimental studies on frailty were excluded from consideration.

### Disability Assessment

This study used the basic activities of daily living (BADL) and instrumental activities of daily living (IADL) scales to assess disability. The BADL scale assesses six abilities: feeding, transfer, walking indoors, dressing, bathing, and toileting (Katz, Ford, Moskowitz, Jackson, & Jaffe, 1963). The IADL scale assesses five abilities, including shopping, going out, food preparation, housekeeping, and doing laundry (Lawton & Brody, 1969).

### Frailty Assessment

This study adopted Cardiovascular Health Study (CHS) indicators because they are commonly employed to evaluate frailty. Proposed by Fried et al. (2001), the indicators evaluate five conditions including grip strength, walk speed, exhaustion, physical activity, and unintentional bodyweight loss. Individuals who attain the score thresholds on three, one to two, or none of the five conditions are categorized as frail, at risk of frailty, and robust, respectively. However, because the CHS indicators involve numerous items and require extensive measurement time, Ensrud et al. (2008) proposed the Study of Osteoporotic Fractures (SOF) indicators, which incorporate fewer items, feature comparable reliability and validity, take less time, and are easier to use.

The SOF indicators assess frailty using three conditions, including unintentional weight loss of more than 5% over the past year, inability to rise five times from a chair without using armrests, and answering “no” to the question “Do you feel full of energy?” People who experienced three, one to two, or none of the aforementioned conditions are categorized as frail, at risk, and robust, respectively. Conducting assessments in community settings requires the use of quick, simple indicators to effectively and rapidly evaluate frailty and risk of disability in older adults. However, no studies have assessed whether indicators of frailty predict disability in community-dwelling older adults.

### Data Extraction and Quality Assessment

Two researchers collected and examined data independently. We analyzed the data collection methods adopted in the selected studies as well as sample size, prevalence, incidence, and correlations between frailty at various stages and the development of disability. A third data reviewer examined inconsistent analysis results.

The Newcastle–Ottawa Scale was used to assess the cohort studies for selection, comparability, and assessment of outcome/exposure (Wells et al., 2014), with a maximum possible score of 9. Scores ≥ 7 indicated a low risk of bias, scores of 4–6 indicated a moderate risk of bias, and scores < 4 indicated a high risk of bias.

### Data Synthesis

The *HR* and 95% CIs of frailty status groups were extracted in the meta-analysis and combined using a random-effects model. In contrast to fixed-effects models, random-effects models enable the true underlying effect to vary among individual studies and assume that a normal distribution is followed. Summary *HRs* were obtained from subgroups according to the frailty criteria (CHS criteria for frailty vs. SOF criteria for frailty) and compared using a mixed-effects model. The heterogeneity of *HRs* across the studies was evaluated using *I*^2^ statistics to measure the extent of overlap among the 95% CIs of the *HRs* obtained from the individual studies. For the *I*^2^ statistics, values of greater than 25%, 50%, and 75% were considered to indicate low, moderate, and high heterogeneity, respectively (Higgins, Thompson, Deeks, & Altman, 2003). Three potential moderators were identified to explain the causes of heterogeneity using mixed-effects models. The potential moderators included study area (Europe, United States, and other regions), sample size (≤ 2,000 or > 2,000), and length of follow-up duration (≤ 1 or > 1 year). Publication bias was assessed using a funnel plot and was tested using Egger's intercept test (Egger, Davey Smith, Schneider, & Minder, 1997). Data analyses were performed using Comprehensive Meta-Analysis 2.2 (Biostat, Inc., Englewood, NJ, USA).

## Results

### Study Sample

**Figure 1.**
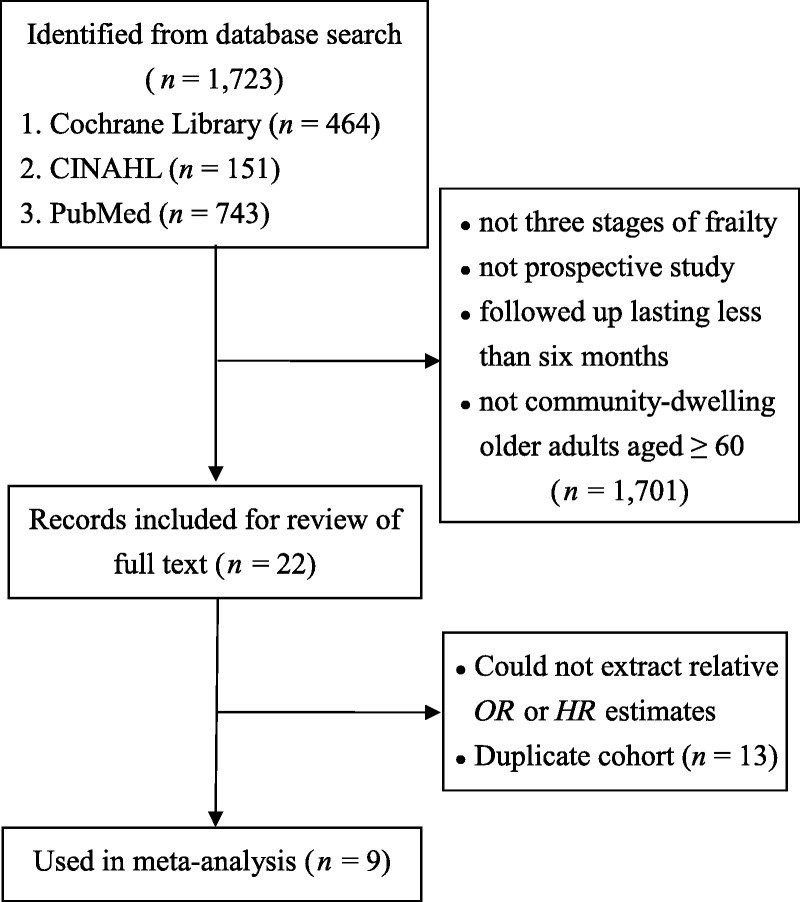
The enrollment of sampled studies. CINAHL = Cumulative Index to Nursing and Allied Health Literature; *OR* = odds ratio; *HR* = hazard ratio.

Figure 1 depicts the details of the literature review. Among the 1,723 studies initially identified, many were excluded because of missing data (*HR* or odds ratio), including inpatient or nonelderly populations, or using duplicate cohorts. Nine prospective cohort studies were identified and mutually agreed on by the two reviewers.

Table 1 summarizes the characteristics of the studies included in the meta-analysis. In total, the samples comprised 32,998 participants and recorded 8,666 disabilities, with a mean follow-up time of 30.4 months (*SD* = 29.26).

**TABLE 1.**
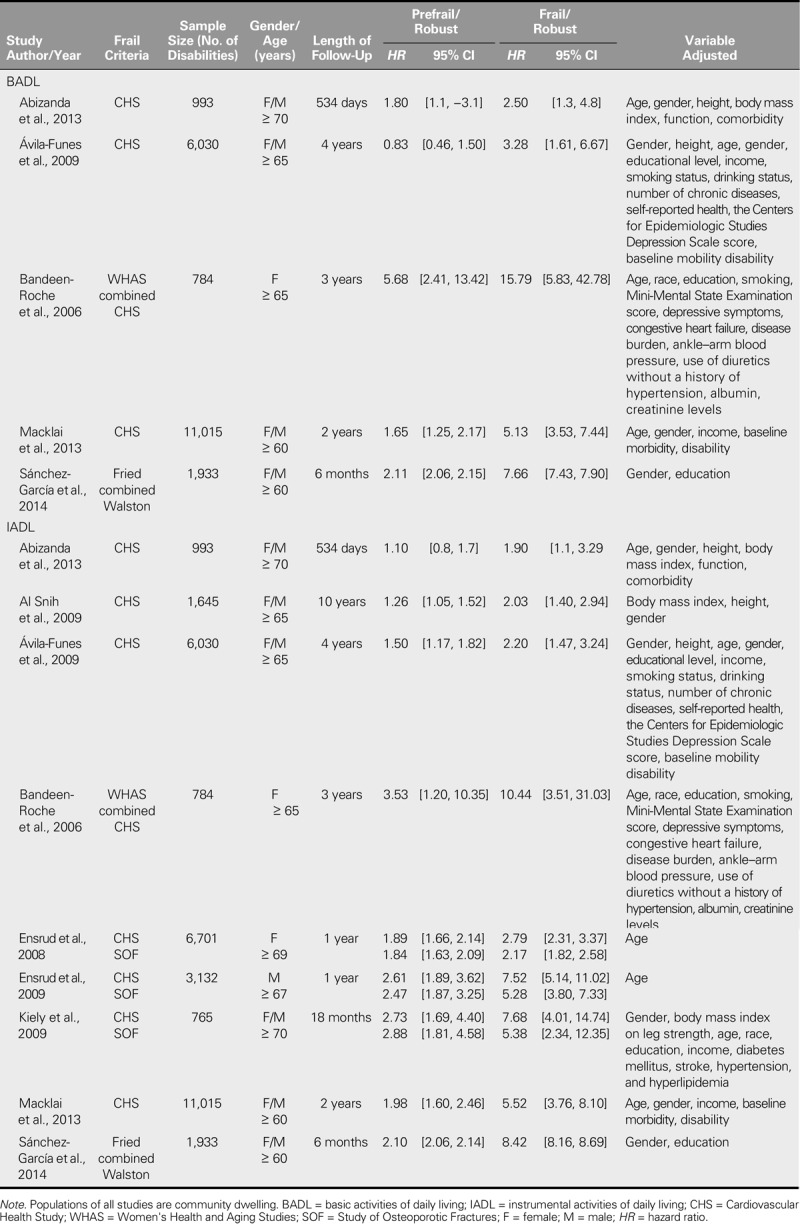
Details of Studies Assessed as Suitable for Inclusion

All of the studies considered IADL, and five also examined BADL. None of the studies reported SOF data. Therefore, the subgroup analysis of frailty criteria could not be performed for BADL disability. Regarding IADL disability, three of the studies provided both CHS and SOF data (Ensrud et al., 2008, 2009; Kiely et al., 2009) and four studies provided CHS data (Abizanda et al., 2013; Al Snih et al., 2009; Ávila-Funes et al., 2009; Macklai et al., 2013).

### Quality Assessment

Study quality was assessed using appropriate items from the Newcastle–Ottawa Scale. Most studies presented a low risk of bias, and most were classified as having representative samples (Table 2).

**TABLE 2.**
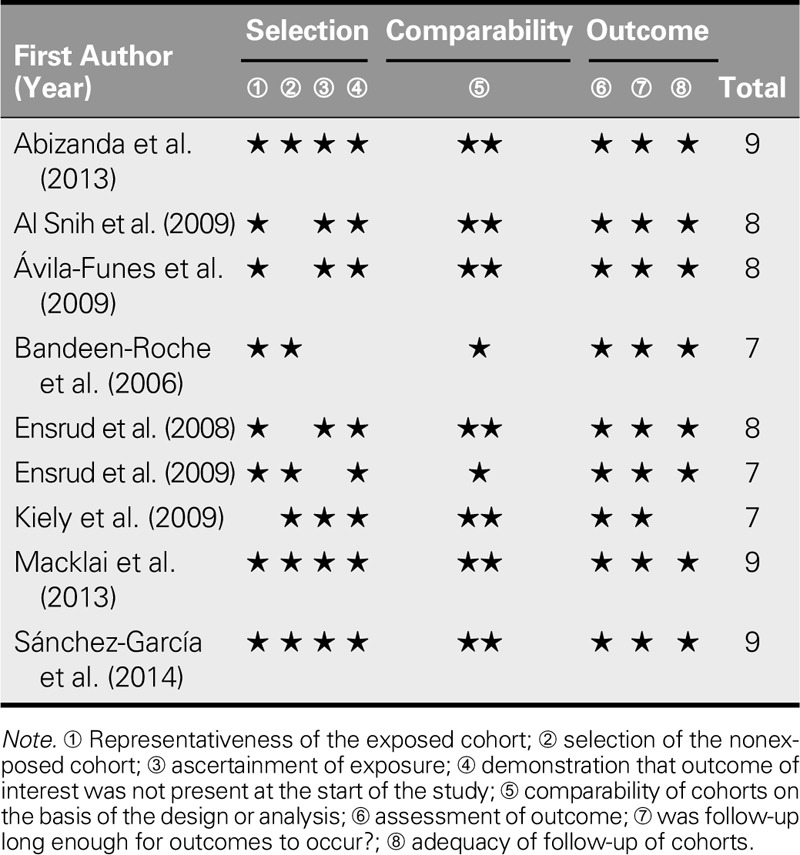
Newcastle–Ottawa Scale Quality Assessment of Included Studies

### Comparing Frailty Groups According to Basic Activities of Daily Living

**Figure 2.**
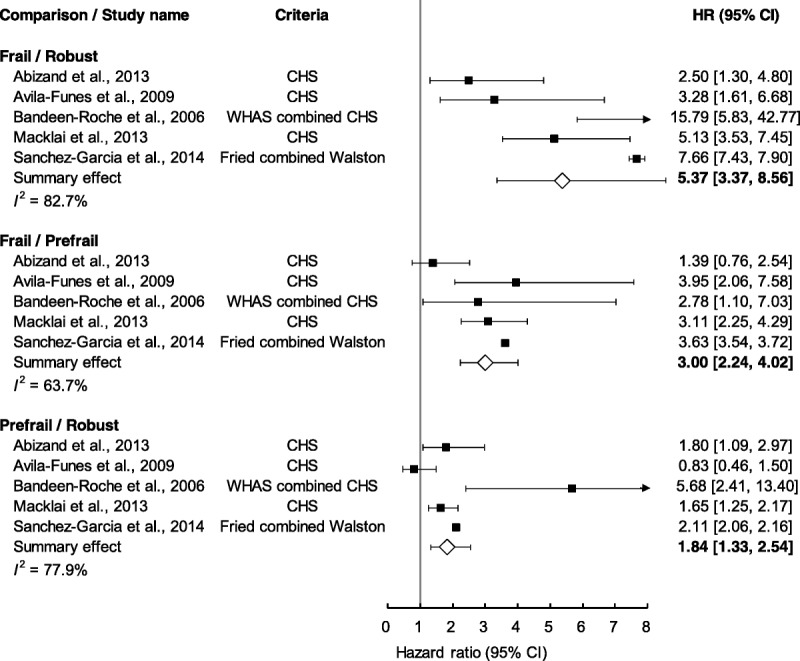
Summary estimates on the risk of “basic activities of daily living” disability. CHS = Cardiovascular Health Study; WHAS = Women's Health and Aging Study; *HR* = hazard ratio; CI = confidence interval.

Figure 2 presents a summary comparison of the different groups by degree of frailty according to BADL disability criteria. The square-shaped filled symbol located on the lines indicates the *HR* of the individual studies, whereas the diamond-shaped unfilled symbol represents the summary *HR* of all the studies. The results show that the level of BADL disability for the frail group was higher than those for the robust group (summary *HR* = 5.37, 95% CI [3.37, 8.56]) and for the at-risk group (summary *HR* = 3.00, 95% CI [2.24, 4.02]). The level of BADL disability in the at-risk group was higher than that in the robust group (summary *HR* = 1.84, 95% CI [1.33, 2.54]).

### Comparing Frailty Groups According to Instrumental Activities of Daily Living

**Figure 3.**
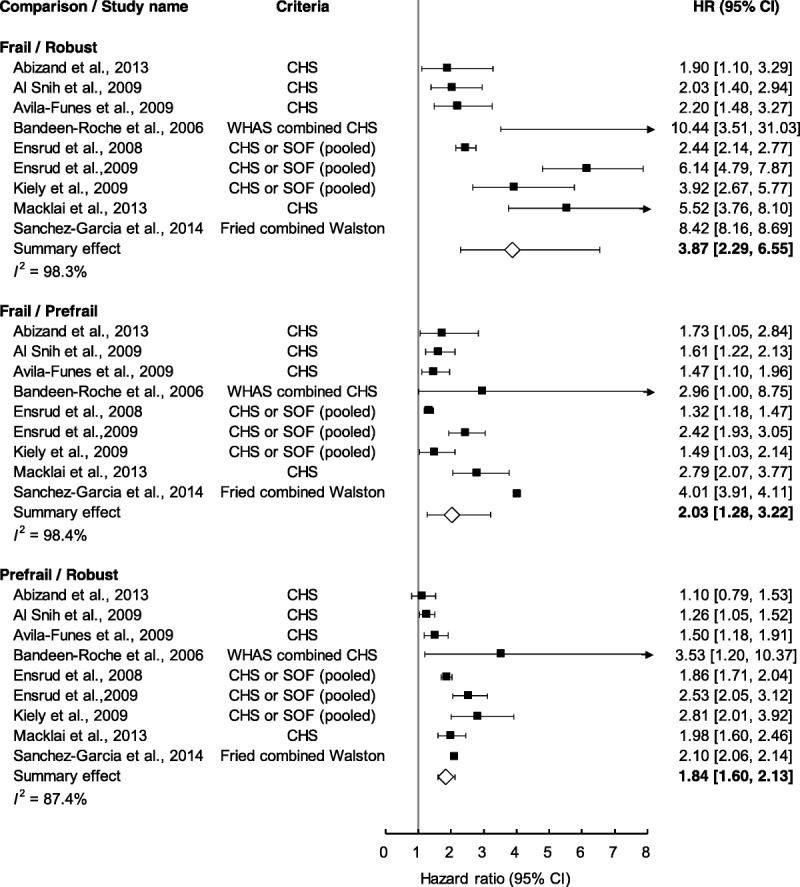
Summary estimates on the risk of “instrumental activities of daily living” disability. CHS = Cardiovascular Health Study; SOF = Study of Osteoporotic Fractures; WHAS = Women's Health and Aging Study; *HR* = hazard ratio; CI = confidence interval.

Figure 3 displays the summary comparison of different groups by degree of frailty according to IADL disability criteria. The level of IADL disability for the frail group was higher than those for the robust group (summary *HR* = 3.87, 95% CI [2.29, 6.55]) and for the at-risk group (summary *HR* = 2.03, 95% CI [1.28, 3.22]). The level of IADL disability for the at-risk group was higher than that for the robust group (summary *HR* = 1.84, 95% CI [1.60, 2.13]).

### Subgroup Analysis of Frailty Criteria: Cardiovascular Health Study vs. Study of Osteoporotic Fractures

**Figure 4.**
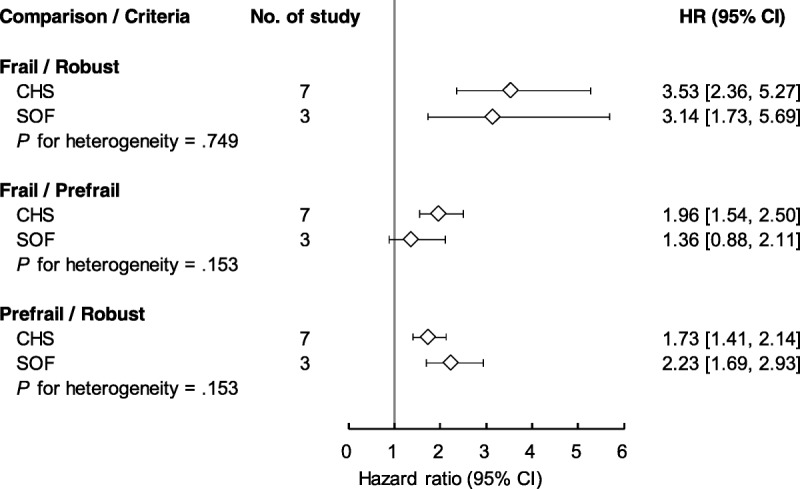
Comparing groups of frailty status subgrouped by CHS and SOF on the risk of “instrumental activities of daily living” disability. CHS = cardiovascular Health Study; SOF = Study of Osteoporotic Fractures; *HR* = hazard ratio; CI = confidence interval.

Figure 4 compares the levels of IADL disability according to CHS and SOF frailty criteria. The summary *HRs* for CHS and SOF were 3.53 (95% CI [2.36, 5.27]) and 3.14 (95% CI [1.73, 5.69]), respectively, in the frail and robust groups. For the frail and at-risk groups, the summary *HRs* for CHS and SOF were 1.96 (95% CI [1.54, 2.50]) and 1.36 (95% CI [0.88, 2.11]), respectively. The summary *HRs* for comparing the at-risk and robust groups were 1.73 (95% CI [1.41, 2.14]) and 2.23 (95% CI [1.69, 2.93]) for CHS and SOF, respectively. Notably, the summary *HRs* obtained using the CHS and SOF criteria did not differ significantly in these comparisons.

### Heterogeneity and Publication Bias

The between-study heterogeneity in this meta-analysis was moderate to high (*I*^2^ = 50%–75%) or high (*I*^2^ > 75%). The results of a further analysis indicated that geographic area may be a moderator when contrasting the frail and robust groups (*p* = .003) and the at-risk and robust groups (*p* = .012) on BADL. The study conducted in the United States had a higher *HR* than studies conducted in other areas (Table 3). Likewise, the results showed that *HRs* obtained from different geographic areas were significantly different when contrasting the frail and robust groups (*p* < .001) and the frail and at-risk groups (*p* < .001) for IADL. The study conducted in Mexico had a higher *HR* than studies conducted in other areas (Table 3).

**TABLE 3.**
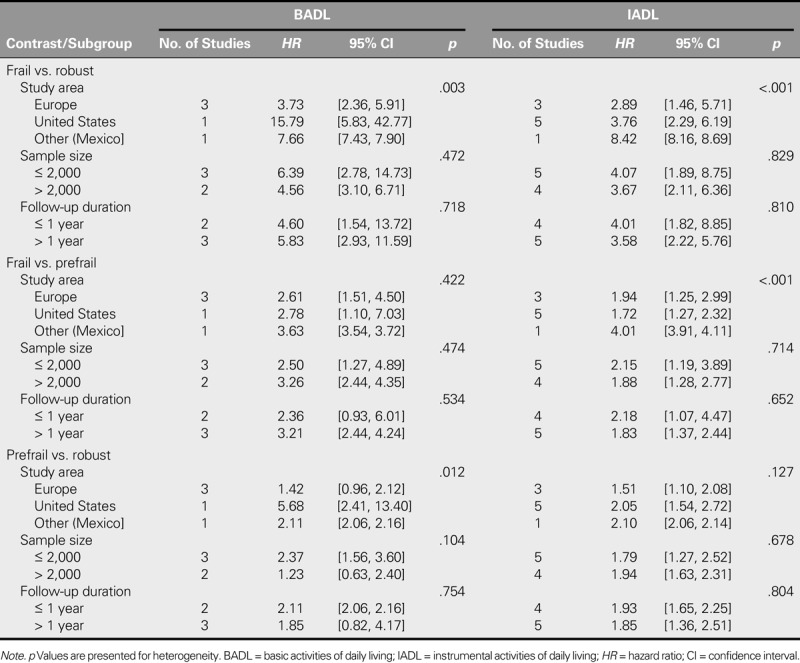
Subgroup Analysis of BADL and IADL

The shape of the funnel plots for the outcome of BADL disability resembles a symmetrical pattern, and the Egger's test results were not significant, suggesting no apparent publication bias. However, a threat of publication bias was observed in a comparison of frail groups with other groups according to IADL disability (frail vs. robust, *p* = .053; frail vs. at-risk, *p* = .040). The funnel plots were examined, and one study (Bandeen-Roche et al., 2006) was found to be an outlier containing a wide CI for its *HR*, suggesting a rare event number or small study size. However, the conclusion regarding IADL disability remained the same when this study was removed, and the problem of publication bias was no longer present.

## Discussion

The WHO (2002) identified frailty prevention as a crucial indicator of successful aging. However, no previous meta-analysis has examined the influence of frailty on disability in older adults to identify the correlation between frailty and disability. To our knowledge, this is the first study to conduct a systematic review and meta-analysis to investigate the relationship between frailty and disability in community-dwelling adults aged 60 years or older.

This meta-analysis found that frail older adults face a higher risk of disability than robust older adults. The current results are in accord with previous studies, which identified frailty as the main risk factor for disability in older adults (Abizanda et al., 2013; Al Snih et al., 2009; Ávila-Funes et al., 2009; Bandeen-Roche et al., 2006; Ensrud et al., 2008, 2009; Kiely et al., 2009; Macklai et al., 2013; Sánchez-García et al., 2014). These studies presented inconsistent results regarding the relative risks of disability faced by frail, at-risk, and robust older adults, with some studies indicating a progressively greater risk (Al Snih et al., 2009; Ensrud et al., 2008, 2009; Kiely et al., 2009; Macklai et al., 2013; Sánchez-García et al., 2014). Others indicated no significant difference in level of risk among these groups (Abizanda et al., 2013; Ávila-Funes et al., 2009; Bandeen-Roche et al., 2006; Kiely et al., 2009).

This evidence-based study, which used BADL and IADL criteria to assess disability, found that frail older adults face the highest risk of disability, followed by at-risk and robust peers. Research indicates that older adults with frailty typically experience a decline in overall function and in specific functions of multiple organs, which marks the beginning of functional deterioration. Moreover, frail older adults easily develop related complications. Therefore, once older adults develop frailty or exhibit signs of risk, they face significantly higher probability of falls, becoming dependent, and becoming disabled, which may eventually lead to hospitalization, institutionalization, and death (Chang & Lin, 2015). Previous studies have argued that disability associated with frailty increases the burden on the individual, caregivers, and society in general (Freiheit, 2015). The early assessment of frailty in older adults should be prioritized for early and effective interventions (e.g., resistance exercise, nutritional supplements) that improve robustness and reduce the incidence of disability.

The studies included in this meta-analysis assessed frailty using various criteria including muscle mass, muscle performance, and muscle strength and effectively predicted the influence of the biological phenotype of frailty in older adults on disability. No difference was found between the CHS and SOF results in identifying frailty or disability; both may be used to effectively determine the correlations between various levels of frailty and disability.

The average follow-up period was 30.4 months, ranging from 6 months (Sánchez-García et al., 2014) to 10 years (Al Snih et al., 2009). The variance likely resulted from differences in participant ages and from data collection methods. Sánchez-García et al. investigated community-dwelling people aged 60 years and older in Mexico, visiting the participants to assess disability levels with a follow-up period of 6 months. Al Snih et al. conducted a prospective cohort study of 1,645 Mexican Americans aged 67 years or older. Because frailty gradually causes disability, older adults may become unable to eat, move, walk, dress, bathe, or use the toilet. Therefore, a 10-year follow-up period was used to observe the development of disability in participants.

This study has several strengths. To the best knowledge of its authors, this study was the first to apply systematic review and meta-analysis to analyze the association between frailty and disability in community-dwelling adults aged 60 years or older. Therefore, the results of this study provide a reference for assessing and treating frailty in community-dwelling older adults in the future.

This study also has several limitations. First, according to the meta-analysis, a medium to high level of heterogeneity existed among the studies. Therefore, generalizing the findings of this study should be done only with appropriate caution. Second, the studies used disparate criteria to define frailty. Although the subgroup analysis revealed that frailty in older adults may result in disability (despite the disparate criteria used), statistical errors may have occurred in the subgroup analyses. Moreover, the follow-up periods in the reviewed studies varied considerably, ranging from 6 months to 10 years, which may have affected how the risk of disability was estimated. Finally, regarding the prediction of risk for disability, although confounding variables were controlled in most of the studies, the number and content of the adjusted variables differed, which may influence the consistency of estimates. Despite these potential limitations on the results of the meta-analysis and the causal inferences in this study, the current findings offer a reference for healthcare professionals to assess the risk of disability in older adults with frailty. In addition, the results of this study provide a foundation for healthcare decision making and assessment.

### Conclusions

Frailty is an important healthcare issue for older persons with geriatric syndromes. Frailty-associated disability severely affects individuals and their families. Early assessment and prevention of frailty hold the potential to reduce the incidence of disability. Nursing professionals should assess frailty in older adults. In addition, methods for effectively preventing frailty in older adults (e.g., progressive resistance exercise, nutrition consultation) should be developed to further reduce the incidence of disability in this population and to improve quality of life.
